# Family Social Environment and Parenting Predictors of Alcohol Use among Adolescents in Lithuania

**DOI:** 10.3390/ijerph14091037

**Published:** 2017-09-08

**Authors:** Linas Šumskas, Apolinaras Zaborskis

**Affiliations:** Faculty of Public Health, Lithuanian University of Health Sciences, A. Mickevičiaus Street 9, LT-44307 Kaunas, Lithuania; apolinaras.zaborskis@lsmuni.lt

**Keywords:** adolescents, alcohol use, family environmental, parenting style, intact family

## Abstract

The role of the family as the social environment in shaping adolescent lifestyle has recently received substantial attention. This study was focused on investigating the association between familial and parenting predictors and alcohol use in school-aged children. Adolescents aged 13- and 15-year from a representative sample (*N* = 3715) of schools in Lithuania were surveyed during the spring of 2014. The methodology of the cross-national Health Behaviour in School-aged Children (HBSC) study was applied. HBSC international questionnaires were completed in the classroom anonymously for obtaining information about drinking of alcoholic beverages and family characteristics—family’s affluence and structure, style of communication in the family, parenting style, parental monitoring, family time together, etc. Univariate and multivariate logistic regression analysis was applied for assessment of the association between familial variables and weekly alcohol use. Analysis has demonstrated that adolescents from non-intact families tended to show significantly higher risk of being weekly drinkers (OR = 1.69; 95% CI: 1.30–2.19). The following parenting factors were associated with weekly use of alcohol: father’s and mother’s low monitoring, father’s authoritarian-repressive and mother’s permissive-neglectful parenting style. Frequent family time together and frequent electronic media communication with parents showed an inverse negative effect than was predicted. The study suggests that alcohol misuse among adolescents could be associated with a non-intact family structure as well as with complex family and parenting determinants which should be investigated more thoroughly by further studies.

## 1. Introduction

Alcohol is one of the most important ill health determinants in the EU and on the global scale [1,2]. The potential risk of alcohol consumption to the health of young generations is demonstrated by the multiple morbidity, mortality and other international studies [3,4]. Young school students between the ages of 12 and 19 are more likely to drink alcohol rather than smoke cigarettes or use illicit drugs, including marijuana [5]. Although research observations indicate that modern adolescents tend to be exposed quite frequently to binge drinking—they consume considerably more alcohol per occasion (five drinks or more on average)—such alcohol use is particularly harmful for underage youth [6].

Multiple observation data demonstrate that in many cases, population-based strategies which have focused their activities on general measures, such as increases of alcohol prices or ban on advertising, have their limitations [7]. This indicates that multiple health settings, including the community, school and family should be taken into account [8].

The family and its structure plays an important role in learning, encouraging and establishing adolescent health behaviour-related values and norms [9]. Children in biological two-parent families show more positive examples of healthy behaviour than adolescents in single-mother, cohabiting stepfather or other type of non-intact families. The adverse effects of marital disruption on child risk-taking behaviours are documented in several research studies [10,11]. Different research studies, which were focused on the parenting related issues has identified that parental warmth and support, also especially communication with parents, have a significant influence on adolescents’ development and health behaviours [12,13].

The societal transition in Central and Eastern Europe, including Lithuania, has resulted in significant demographical and social changes during the last few decades. These changes have resulted in a decline of birth rates and increased emigration of the young people population. The understanding about the traditional (intact) family and less prevalent non-intact family models (single mother or single father families, etc.) has also shifted. Recent statistics show that the proportions of children living in a traditional nuclear family (with their biological father and mother) have been shrinking in the past decade [14,15,16]. A new generation of children is exposed to these transformations that influence their socialization. Also, research studies have accumulated more and more evidence that the non-intact family is less efficient at controlling the risk-taking behaviours such as smoking, alcohol use and drug abuse [17]. This is why we focused our study not only on showing the family determinants of alcohol use in children, but also on highlighting the protective factors associated with the interpersonal relationships and parenting style, which could be applied during alcohol prevention programmes in youth.

A review of the related literature shows that better control and adjustment are achieved in children within families where more open communication with their parents was practiced. In addition, closer relations in the family, including physical presence and emotional support, were proved to play a protective role in prevention of alcohol abuse [8,17,18]. Even though research on family influences on adolescent behaviour outcomes has expanded during the last few decades, we still see many gaps in this field of research. The international multicentre HBSC study team and its Family Culture working group have developed standardised questionnaires and suggested an optional package on the family. This allowed us to conduct research and get deeper insights into family influences on health behaviour in a sample of Lithuanian adolescents [19].

The aim of this research paper was to investigate the association between alcohol use and a range of familial factors in a representative sample of Lithuanian adolescents who were surveyed in the HBSC survey 2014. We presupposed that changes in family structure, weaker child-parent relationships and communication, shortage of parental control, etc., would be related to higher risk of alcohol use in adolescents.

## 2. Methods

### 2.1. Subjects and Study Procedures

The data on health behaviour analysed below were collected in a school-based, cross-sectional, anonymous survey conducted during April–May 2014 in Lithuania in the framework of the World Health Organization collaborative cross-national HBSC study [20]. Team of researchers followed the standardized international research protocol to ensure consistency in survey instruments, data collection and processing procedures [21].

Secondary school students aged 11-, 13- and 15-years were invited to participate in the questionnaire survey. Clustered hierarchical sampling was used to form the pool of respondents. The initial sampling unit was a class of students of the 5th, 7th or 9th grades. According to the research protocol, students selected were representative by gender and age. Also, the recommendation to cover 1500 or more students per age group size for the national sample was followed. In total, 129 schools with 356 classes from Lithuania were included to ensure the necessary number of surveyed students.

Questionnaire forms were distributed in school classrooms by the form tutors who followed with written instructions. One-two schooling periods (45 min each) were provided as the time frame for filling out the questionnaires. Eligible participants could freely choose to participate or not in the survey. Measures of anonymity and confidentiality were ensured. Students sealed the provided envelopes with the questionnaires inside after finishing answering. Class tutors were instructed about the process of carrying out the survey and how to report the number of participants.

After the completion of the questionnaire survey, the data were prepared and exported to the HBSC International Data Bank at the University of Bergen, Norway. The data files were checked, cleaned and included into the international HBSC database. The final Lithuanian data file which includes information on 5730 respondents aged 11-, 13- and 15-year was formed. Thus, our study covered 3715 students aged 13- and 15-year. Among them, the proportion of non-reported frequency of alcohol was 4.2% (*N* = 156) and the proportion of non-reported drunkenness in lifetime was 0.7% (*N* = 26). The youngest group of 11-year-old adolescents (*N* = 2015) was excluded from the analysis because the proportion of respondents who reported regular use of alcohol and frequent drunkenness during their lifetime was relatively low (4.0% and 1.7%, respectively). The response rate in the total sample was 84%.

### 2.2. Instrument and Variables

The HBSC international questionnaire that was adopted after its translation from the Standard English version into Lithuanian and retranslated back into English was applied in our survey [21]. The questionnaire covered a mandatory part and an optional section. The optional package on “Family Culture” was included into the Lithuanian version of the questionnaire [19].

The main outcome variable used in the present study was weekly drinking of any alcoholic beverage. The question posed was: At present, how often do you drink alcoholic beverages: (a) beer; (b) wine; (c) vodka, brandy, whisky or spirits/liquor; (d) champagne or sparkling wine; (e) alcohol mixes (alcopops, cider, Mix, Fizz and similar preparations)? The following answer options were included: ‘every day’; ‘every week’; ‘every month’; ‘rarely’ and ‘never’. Categories ‘every day’ and ‘every week’ were merged in order to create the analysed outcome variable.

The list of independent variables covered such demographical indicators as gender and age group (13-year-olds and 15-year-olds). In addition, family structure variable, Family Affluence Scale (FAS) and a set of 15 familial parenting communication related variables were included: (1) satisfaction with family relationships; (2) communication with father; (3) communication with mother; (4) father’s monitoring; (5) mother’s monitoring; (6) quality of communication with family; (7) school-related parental support; (8) father’s emotional support; (9) mother’s emotional support; (10) father’s parenting style; (11) mother’s parenting style; (12) family time together; (13) electronic media communication with parents; (14) seeing father; (15) seeing mother.

The details on the mentioned familial variables are described in HBSC study publications [20,21] and also presented below:

*Family Affluence Scale (FAS).* A set of six questions on the living standards of the households in which children live were used. The questions covered: (1) car ownership; (2) having a bedroom; (3) having bathroom facilities; (4) having holidays with parents; (5) having a computer; (6) having a dishwashing machine at home [22]. The possible categories for family affluence were suggested as: low (reference group), ‘medium’ and ‘high’.

*Family structure.* The respondents were asked to indicate the individuals living within their family in the list of adult people [21]. Two categories were used in our analysis—intact family (reference group), and non-intact family. Intact family (or biological, two parent family) was coded in cases when respondents reported living with the mother and the father.

*Communication with parents.* Questionnaires included a question ‘How easy it is to talk to your father and mother about things that really bother you?’ [23]. The categories ‘easy’ (reference group) and ‘difficult’ were used in further analysis.

*Quality of communication within the family.* A shortened version (four items) of the clear communication scale from Family Dynamics Measure II was applied [24,25]. Two categories were used in analysis—‘good communication’ (reference group) and ‘poor communication’. Our data analysis showed that this scale has very good reliability (Cronbach’s Alpha was 0.87 in the total sample and the subsample of intact families).

*Satisfaction with family relationships.* This variable was measured by means of an item based on Cantril’s [26] ladder scale, which allowed to rate satisfaction from 0 to 10. Range of 7–10 scores was considered as ‘high satisfaction’ (reference group), 0–6 as ‘low satisfaction’.

*Parental monitoring.* The measure which was developed by Brown et al. was used [27]. This measuring instrument included the question ‘How much does a student’s father and mother know about five issues of the students’ life: (a) who are your friends; (b) how do you spend your money; (c) where are you after school; (d) where do you go at night; (e) what do you do during free time. The answers covered the following range: (a) ‘doesn’t know anything’; (b) ‘knows a little’; and (c) ‘knows a lot’. Higher knowledge represented higher levels of parental monitoring (reference group). In the measurement scale reliability analysis Cronbach’s Alphas were 0.79 and 0.90 respectively for the mother and the father.

*Emotional support.* The evaluation of parental warmth and support was based on the four items subscale of the standard instrument developed by Parker et al. [28]. The respondents were asked ‘how often their mother and father (separately) provide eight different (eight categories included) of support or express parental warmth (e.g., ‘helps me as much as I need’, ‘is loving and balmy’, ‘understands my problems and worries”, etc.). The options of answers were scored as ‘never’, ‘sometimes’, ‘almost always’. The scale reliability analysis showed Cronbach’s Alpha 0.78 for mothers’ and 0.84 for fathers’ answers. Categories for ‘high’ (reference group) and ‘low’ parental emotional support were developed according positive and negative factor score values which were calculated in one-factor analysis of the subscale variables.

*School-related parental support.* The theoretical framework for school-related support was developed by the HBSC team and used in previous surveys [29]. The respondents were asked to what extent they agree or disagree concerning the five statements which are related with the learning process at school, emotional support and learning motivation: (a) ‘If I have a problem at school, my parents are ready to help’; (b) ‘My parents are willing to come to school to talk to teachers’; (c) ‘My parents encourage me to do well at school’; (d) ‘My parents are interested in what happens to me at school’; and (e) ‘My parents are willing to help me with my homework’. A binary variable was created with two categories—”high support” (reference group) and “low support”. Our statistical evaluation showed very good reliability of the school-related parental support scale—Cronbach’s Alpha was 0.85 both in the total sample and the subsample.

*Parenting style.* Questionnaire items according Maccoby and Martin were included [30]. The scale covered four parental styles: (a) authoritative-reciprocal (reference group) (b) authoritarian-repressive; (c) permissive-indulgent; and (d) permissive-neglectful. School students were asked: ‘What does your father/mother does, when you do something that is wrong?’ Four response options (e.g., ‘My father/mother doesn’t punish me, he/she takes no notice’ or ‘My father/mother punishes me immediately without telling me why’, etc.) were offered to choose for respondents. Each category of answers represented the corresponding parental educational style.

*Family time together.* Eight items were used in the questionnaire for evaluation of joint family activities. The following activities were listed: sitting and talking about things together, watching TV or a video together, playing indoor games together, eating meals together, going for a walk together, eating meals together, going places together, visiting friends or relatives together, playing sports together [31]. School students were asked to respond how often they are involved of these joint family activities. Two categories—‘often time together’ (reference group) and ‘rare time together’—were used in further analysis. Family time together scale showed good reliability—Cronbach’s Alpha was 0.85 both in the total sample and the subsample.

*Electronic media communication with parents.* The questionnaire included question about different electronic modes of communication with parents. Four ways of communication were covered: (a) speaking on the phone; (b) sending text messages; (c) speaking in real time on Internet; and (d) writing e-mail letters to parents. Several options of answers were proposed to choose for the respondents: ‘never’, ‘once per week’, ‘several times per week’, ‘once a day’, and ‘several times a day’ [21]. The Cronbach’s Alpha for this scale was 0.61. Respectively, positive and negative factor score values derived from one-factor analysis were used as indicators for ‘often’ (reference group) and ‘rare’ electronic media communication with parents.

*Seeing of mother and father.* The additional optional item on how often children are able to meet or see their parents because of job or other different circumstances was included into the Lithuanian version of HBSC questionnaire. The question was formulated to receive responses about seeing mother and father separately. Five possible responses (from ‘every day’ to ‘haven’t seen for more than a year’) were offered to choose from for the surveyed students. In further analysis five response options were grouped into two categories: (a) seeing father/mother every day, and (b) seeing father/mother not every day.

### 2.3. Statistical Analysis

Two steps analysis was applied. The total sample of 13- and 15-year-olds (*N* = 3715) was investigated during the first stage of the analysis in order to assess the relationship between alcohol drinking behaviour and family structure. The subsample of adolescents living in intact families (*N* = 2542) was analysed in the second step of analysis, which was aiming to explore relationships between weekly alcohol use and a set of mentioned above family and parenting variables. 

Cronbach’s Alpha was used to evaluate the level of internal consistency reliability in the above presented multi-item scales. One-factor explanatory analysis with a principal component analysis was applied for each scale. This method allowed to build the reliable single-dimensional variables. The factor scores were analysed according to factor score based model. The higher factor scores showed higher quality level of parenting practices in the families. In addition, we used 0 as a cut-off point to dichotomize factor score values into positive and negative groups which corresponded to respondents' higher and lower scores in the scale.

Logistic regression analysis was applied in order to investigate associations between familial, parenting determinants and alcohol use. In the first step, we conducted univariable binary logistic regression (BLR) analysis. Next, Enter method was used in the multivariable analysis. Irrespective of their significance found in the first step of the analysis, all variables were included during the second stage. Interactions between familial and parenting variables were also tested. The whole analysis was performed using the Complex Samples module of SPSS (version 20.0, Chicago, IL, USA) which adjusts for the complex cluster-stratified sampling method (schools, classes) and weighted data [32]. *p* < 0.05 was considered to be statistically significant.

### 2.4. Ethical Statement

Ethics approval for the study was provided by the Kaunas Regional Biomedical Research Ethics Committee (reference number BE-2-16). The study conformed to the principles outlined in the World Medical Association’s Declaration of Helsinki. The study was also endorsed by the local educational authorities and the Ministry of Education and Science, Lithuania. Additionally, written informed consent for participation in the questionnaire survey was obtained from parent of school students.

## 3. Results

Table 1 describes demographic and socio-economic characteristics of all studied adolescents and subsample of adolescents living in an intact family. The results show that study sample was quite representative and balanced by gender. About two thirds (62.8%) of respondents have indicated that they lived in the intact families.

In the total merged sample of 13- and 15-year-old adolescents, the prevalence of weekly consumption of alcoholic drinks was 7.7%. It was higher in boys than in girls (11.5% vs. 4.6%; *p* < 0.001), and was increasing from 5.9% to 10.6% (*p* < 0.001) across period of age 13 to 15 years.

Table 2 presents demographic, socioeconomic characteristics as well as answers of respondents about communication with parents, emotional support, parenting styles in the subgroup of the intact family subsample. A large majority of students (86.3%) were satisfied with their family relationships. Some differences in the students’ opinion about mother’s and father’s role in the family life and parenting were established. Our study showed that ‘easy communication’ with father is less prevalent than with their mother (63.1%; vs. 75.9%; *p* < 0.001). Therefore, lower level of paternal monitoring rather than mother’s monitoring was reported in our analysis (49.3% vs. 61.8%; *p* < 0.001). According to the opinion of adolescents, they feel lower emotional support from fathers than from their mothers (57.1% vs. 61.5%; *p* = 0.002). Also, according to the responses of school students, mothers are more likely to demonstrate the authoritative-reciprocal parenting style in comparison with fathers (46.2% vs. 42.2%; *p* < 0.001). The respondents answered that they see mothers every day more frequently than fathers (94.0% vs. 77.4%; *p* < 0.001).

Table 3 shows data on weekly alcohol drinking among 13 and 15-year-old adolescents by gender, age, family affluence and family structure. Weekly drinking was found to be significantly associated with family structure. Students from non-intact families were more likely to be weekly consumers of alcohol (crude OR = 1.63, 95% CI: 1.28–2.09; adjusted OR = 1.69, 95% CI: 1.30–2.19).

Table 4 shows results of univariable and multivariable logistic regression analysis of some possible family and parenting predictors of weekly alcohol drinking. Univariable BLR analysis showed that weekly drinking of alcoholic beverages among adolescents living in an intact family was significantly associated with the set of the following possible familial predictors: low satisfaction with family relationships; poor quality of communication in the family; low both father’s and mother’s monitoring; low mother’s emotional support; low school-related parental support. In addition, authoritarian-repressive and permissive-neglectful parenting style of both parents (also with permissive-indulgent father’s parenting style), often electronic media communication with parents showed higher odd ratios for weekly alcohol use in univariable regression analysis.

Multivariable logistic regression analysis (after adjusting the data for gender, age and family FAS) point out the three possible familial and parenting predictors of weekly alcohol use which were significantly associated with increased risk for weekly alcohol use: low mother’s monitoring (OR = 1.66; 95% CI: 1.06–2.60), authoritarian-repressive father’s parenting style (OR = 2.51; 95% CI: 1.27–4.95); permissive-neglectful mother’s parenting style (OR = 3.46; 95% CI: 1.48–8.09). Therefore, rare family time together (OR = 0.64; 95% CI: 0.42–0.97) and rare communication with parents by phone or computer (OR = 0.47; 95% CI: 0.32–0.69) were found as the protective factors to be involved in alcohol consumption. Frequency of ‘seeing mother or father’ did not demonstrate statistical relation with alcohol consumption. This is why the detailed results on this variable were excluded from the Table 4).

## 4. Discussion

This paper has focussed its scope on some social, family and parenting determinants of alcohol use. Therefore, the main emphasis in our analysis was made on the family setting as the important socialisation arena. As we know, parenting communication and other familial determinants could play an important role in development of youth health and heath behaviour [8,17]. We aimed to investigate the association between alcohol use and a set of familial factors, which constitute an important component in the multicourse aetiology of alcohol use among adolescents. The problem of alcohol consumption was selected as an important health issue due to significant scale of this risk-taking behaviour among adults and also among Lithuanian adolescents in recent decades [33].

The study has established that only 62.8% of the total sample of school children were living in the intact families in 2014. The Statistics Lithuania data shows that two decades ago, in 1994, during the first HBSC cross-sectional study in Lithuania, the corresponding figure was significantly greater—82.7% [16]. In addition, one of our main findings was the fact that adolescents from non-intact families had significantly higher risk to be weekly alcohol drinkers (OR = 2.13; 95% CI: 1.78–2.54). Both findings mentioned above (living in non-intact family and its relation with excessive alcohol use) should imply that social context has been deteriorating during the last few decades and this facilitates more frequent alcohol consumption. The results of our study were in accordance with the literature indicating that adolescents from non-intact families were more likely to use alcohol more often than their peers who lived in an intact family [18].

In our study we have demonstrated that despite the fact alcohol use was more common among boys [33,34], the association (alcohol use vs. intact/non-intact family) was equally strong both for boys and girls. It is important to mention, that in 1994 and later surveys of our national HBSC studies the association between weekly alcohol drinking and family composition was much weaker than it was established in 2014 [33]. The presented change in the strength of association could indicate on some processes of life style shifts in the families. Specifically, it could be related to the ongoing societal transition period, during which the percentage of intact families was decreasing in Lithuania [16]. We hypothesize that such change of association could be the result of more significant alteration in the intact and non-intact family functioning and in communication patterns in our society.

The associations between family affluence, family culture, parenting style, and adolescent risk-taking behaviour also have been extensively investigated by researchers [10,35,36]. Different variables to describe family functioning and parenting have been suggested to include as possible family determinants of addictive behaviours. However, in our study we did not establish statistical relation of weekly alcohol consumption with FAS. This is why we focussed our scope on the range of 15 “more soft” possible family and parenting related determinants, which measure different aspects of child-parent relationships. In order to avoid the effect of overestimation of the father’s or mother’s role in the single-parent and step-parent families, a “traditional” intact (or nuclear) family was selected as a model for further analysis of selected parenting style and family culture variables [37].

It was shown in our study that strict father’s and mother’s monitoring, adolescent’s satisfaction with family relations, proper communication, school related parental support play the protective role in controlling alcohol use in youth. The protective role of parental monitoring in prevention of risk- taking behaviours among adolescents has been widely discussed [8]. Several studies have identified that both paternal and maternal monitoring act as a strong protective measure against outcomes of risk-taking behaviours [38]. In line with these studies, our study also supported such findings related with alcohol use. In addition, the current study stresses the importance of mother’s monitoring role in prevention of alcohol use among adolescents.

On the another, hand we did not find in our study that ‘easy communication’ with father or mother as well as ‘seeing mother or father every day’ could have some relations with weekly alcohol drinking. In line with other studies [13,39], the present study showed that adolescents could talk more easy to their mother rather than to their father. This could indicate that mothers could play more prominent role in preventing onset of alcohol use by educating rather than punishing in contrast to more prevalent father’s authoritarian parenting style.

In our research, we made the assumption that frequent interactions with parents on the phone or by other electronic media communication (such as e-mail or texting on the mobile, etc.) can facilitate more positive health behaviour outcomes and play a protective role in alcohol use initiation [19]. However, surprisingly, the results of our study showed the opposite: frequent communication with parents through electronic media increased the risk of weekly drinking almost twice. These finding implying and allow us to hypothesize that electronic media communication could be considered by adolescents as a mode of more distanced formal communication which allows them to escape from closer parents’ supervision. Therefore, our such conclusion needs further, more detailed and thorough research.

Our study has a number of strengths and also some limitations. First of all, the main strength is related with the large sample of the school student population surveyed, and also with the high response rate and representativeness of the national sample which was selected in accordance with epidemiological study standards. It is also important to mention that our research was conducted in the framework of the internationally well-known cross-national collaborative HBSC study. This allowed to apply standardized methods, approved by experts including the HBSC questionnaire which was developed by HBSC team. The evaluation of family environment and parenting indicators were based on validated scales. The analysis showed the high coefficients of *Cronbach*’s alpha internal consistency reliability in all applied scales. These results of our study has provided additional clarifications and filed some gaps in social research on consequences of parenting on adolescent outcomes related with risk taking behaviour.

Some limitations of our study are related to an intrinsic constructive problem of our methods used. Questionnaire surveys use self-reported data, which could be a potential source of bias [40,41,42]. To avoid this kind of a potential bias of self-reporting, special procedures aiming to provide and warranty the confidentiality and anonymity and were used. Piloting and pre-testing of questions before the main survey also was implemented as a routine procedure at the local and international level. The cross-sectional design of this study also constitutes a limitation that does not allow establishing the causality. This issue is very common when we analyse only a statistical relation between potential cause and outcome in the cross sectional studies. Thus, more studies, including studies with a longitudinal design, are needed to confirm the results and to establish scientific evidence on the relationships found in this study. In addition, it is evident that future research should involve also larger number of contextual and even genetic factors [43].

In our study, we also assessed the affluence of the family. Such evaluation was based mainly on gathering information on a family’s material items and commodities This may lead to misclassification of some families that are affluent, but that save rather than spend. Only one dimension of family’s socio-economical and educational status SES was examined, whereas in other studies authors also have included parents’ education or occupation as important variables of analysis.

Data analysis approach that was used in our research, was focussed on the analysis of associations between weekly alcohol use and familial variables within intact families only. The reasons for such analytical approach were related to some methodological constraints we faced. This was mainly related with the limited application of some questions for mother and father both for non-intact and intact families. The described methodological issue requires further more detailed investigation of family and parenting predictors of alcohol use also in different types of non-intact families—in single-parent families, step-father families, cohabiting parent families, grandparent and primary caregiver families.

## 5. Conclusions

Family environments and parenting practices constitute the foundation for further development of healthy lifestyle during adolescence. Our study showed that parenting practices show a wide variance. The multiple and rich results of the study cannot be explained easily also because of complex nature of alcohol use in adolescents. With some exceptions the majority of our findings are in line with our hypothesis and also confirm the findings of other researchers. The results of study suggest that prevalence of weekly alcohol use among adolescents of Lithuania is associated with a non-intact family structure as well as weaker parental support. In addition, maternal monitoring seems is a particularly important as the main protective factor. At the same time, it seems that the role of the father in parenting is diminishing in our changing society. The results of our study demonstrate that family environment and parenting practices are critical components to be incorporated into prevention programmes on prevention of alcohol use in youth.

## Figures and Tables

**Table 1:** Demographic and social characteristics of the total sample and subsample studied.

Independent Variables	*n*	%
Total sample studied, *N* = 3715		
**Gender:**		
Boys	1902	51.2
Girls	1813	48.8
**Age:**		
13-year-old	2017	54.3
15-year-old	1698	45.7
**Family FAS:**		
Low	1328	36.9
Medium	1575	43.7
High	700	19.4
**Family structure:**		
Intact family	2542	68.6
Non-intact family	1163	31.4
**Subsample of respondents living in an intact family, *N* = 2542**		
**Gender:**		
Boys	1301	51.2
Girls	1241	48.8
**Age:**		
13 years old	1402	55.2
15 years old	1140	44.8
**Family FAS:**		
Low	784	31.8
Medium	1116	45.2
High	569	23.0

**Table 2:** Responses of the respondents from the intact family subsample about the communication in the family and parenting style of the mother and the father (*N* = 2542).

Predictors	*n*	%	*p* ^a^
**Satisfaction with family relationships:**			
High	2156	86.3	
Low	343	13.7	
**Communication with father:**			
Easy	1494	63.1	<0.001
Difficult	872	36.9	
**Communication with mother:**			
Easy	1801	75.9	
Difficult	573	24.1	
**Quality of communication in the family:**			
Good	1578	62.6	
Poor	944	37.4	
**Father’s monitoring:**			
High	1243	49.3	<0.001
Low	1279	50.7	
**Mother’s monitoring:**			
High	1559	61.8	
Low	963	38.2	
**School-related parental support:**			
High	1303	51.7	
Low	1219	48.3	
**Father’s emotional support:**			
High	1452	57.1	0.002
Low	1090	42.9	
**Mother’s emotional support:**			
High	1563	61.5	
Low	979	38.5	
**Father’s parenting style:**			
Authoritative-Reciprocal	1051	42.2	<0.001
Permissive-Indulgent	1024	41.2	
Authoritarian-Repressive	195	7.8	
Permissive-Neglectful	218	8.8	
**Mather’s parenting style:**			
Authoritative-Reciprocal	1156	46.2	
Permissive-Indulgent	1089	43.5	
Authoritarian-Repressive	159	6.4	
Permissive-Neglectful	98	3.9	
**Family time together:**			
Often	1177	46.7	
Rare	1345	53.3	
**Electronic media communication with parents**			
Often	1099	43.5	
Rare	1428	56.5	
**Seeing of the father**			
Every day	1923	77.4	<0.001
Not every day	562	22.6	
**Seeing of the mother**			
Every day	2346	94.0	
Not every day	149	6.0	

^a^ Significant difference in respondents’ responses about communication in the family with father and mother (Chi-squared test).

**Table 3:** Weekly drinking of alcohol among 13 and 15-year-old adolescents by gender, age, family affluence and family structure (total sample, *N* = 3715): results of univariable and multivariable logistic regression.

Predictors	Non-Weekly Drinking *n* (%)	Weekly Drinking *n* (%)	Univariable Logistic Regression		Multivariable Logistic Regression	
			OR	CI	OR	CI
**Total:**	3272 (92.3)	287 (7.7)				
**Gender:**						
Boys (reference group)	1599 (88.5)	207 (11.5)	1.00		1.00	
Girls	1673 (95.4)	80 (4.6)	**0.37**	**0.28–0.48**	**0.39**	**0.29–0.51**
**Age:**						
13-year-old (reference group)	1819 (94.1)	115 (5.9)	1.00		1.00	
15-year-old	1453 (89.4)	172 (10.6)	**1.87**	**1.46–2.39**	**1.89**	**1.46–2.44**
**Family FAS:**						
Low (reference group)	1159 (91.8)	103 (8.2)	1.00		1.00	
Medium	1403 (92.4)	115 (7.6)	0.92	0.70–1.22	0.97	0.73–1.29
High	618 (91.7)	56 (8.3)	1.02	0.73–1.43	1.17	0.82–1.66
**Family structure:**						
Intact family (reference group)	2273 (93.2)	167 (6.8)	1.00		1.00	
Non-intact family	992 (89.3)	119 (10.7)	**2.13**	**1.78–2.54**	**1.69**	**1.30–2.19**

Significant relationships are provided in bold. OR—Odds Ratio; 95% CI—95% Confidence Interval.

**Table 4:** Possible familial predictors of weekly alcohol drinking among 13 and 15-year-old adolescents subsample (*N* = 2542) of respondents living in the intact family: results of univariable and multivariable logistic regression.

Predictors	Non-Weekly Drinking *n* (%)	Weekly Drinking *n* (%)	Univariable Logistic Regression		Multivariable Logistic Regression ^a^	
			OR	CI	OR	CI
**Gender:**						
Boys (reference group)	1121 (90.3)	121 (9.7)	1.00		1.00	
Girls	1152 (96.2)	46 (3.8)	**0.37**	**0.26–0.53**	**0.39**	**0.25–0.60**
**Age:**						
13-year-old (reference group)	1282 (95.1)	66 (4.9)	1.00		1.00	
15-year-old	991 (90.8)	101 (19.2)	**1.98**	**1.44–2.73**	**2.33**	**1.58–3.44**
**Family FAS:**						
Low (reference group)	703 (93.7)	47 (6.3)	1.00		1.00	
Medium	1005 (93.3)	72 (6.7)	1.07	0.73–1.57	1.34	0.84–2.12
High	505 (92.7)	40 (7.3)	1.19	0.77–1.83	1.52	0.90–2.57
**Satisfaction with family relationships:**						
High (reference group)	1946 (93.6)	132 (6.4)	1.00		1.00	
Low	293 (90.7)	30 (9.3)	**1.51**	**1.00–2.29**	0.84	0.47–1.50
**Father’s monitoring:**						
High (reference group)	1130 (94.9)	61 (5.1)	1.00		1.00	
Low	1127 (91.5)	105 (8.5)	**1.73**	**1.25–2.39**	1.52	0.95–2.44
**Mother’s monitoring:**						
High (reference group)	1435 (92.3)	71 (4.7)	1.00		1.00	
Low	822 (89.6)	95 (10.4)	**2.34**	**1.70–3.21**	**1.66**	**1.06–2.60**
**Quality of communication in the family:**						
Good (reference group)	1421 (94.2)	87 (5.8)	1.00		1.00	
Poor	836 (83.6)	79 (8.6)	**1.54**	**1.13–2.12**	1.29	0.82–2.03
**School-related parental support:**						
High (reference group)	1180 (94.2)	73 (5.8)	1.00		1.00	
Low	1077 (92.1)	93 (7.9)	**1.40**	**1.02–1.92**	0.94	0.61–1.46
**Father’s emotional support:**						
High (reference group)	1303 (93.7)	87 (6.3)	1.00		1.00	
Low	970 (92.4)	80 (7.6)	1.24	0.90–1.69	0.81	0.51–1.30
**Mother’s emotional support:**						
High (reference group)	1413 (94.5)	83 (5.5)	1.00		1.00	
Low	860 (91.1)	84 (8.9)	**1.66**	**1.21–2.28**	1.06	0.67–1.69
**Father’s parenting style:**						
Authoritative-Reciprocal (reference group)	968 (95.5)	46 (4.5)	1.00		1.00	
Permissive-Indulgent	913 (93.4)	65 (6.6)	**1.50**	**1.02–2.21**	1.36	0.84–2.19
Authoritarian-Repressive	170 (89.5)	20 (10.5)	**2.48**	**1.43–4.29**	**2.51**	**1.27–4.95**
Permissive-Neglectful	184 (86.4)	29 (13.6)	**3.32**	**2.03–5.42**	1.96	0.96–4.00
**Mother’s parenting style:**						
Authoritative-Reciprocal (reference group.)	1051 (94.6)	60 (5.4)	1.00		1.00	
Permissive-Indulgent	981 (93.8)	65 (6.2)	1.16	0.81–1.67	1.29	0.83–2.02
Authoritarian-Repressive	138 (89.0)	17 (11.0)	**2.16**	**1.22–3.80**	1.07	0.49–2.36
Permissive-Neglectful	72 (77.4)	21 (22.6)	**5.11**	**2.94–8.87**	**3.46**	**1.48–8.09**
**Family time together:**						
Often (reference group)	1041 (92.3)	87 (7.7)	1.00		1.00	
Rare	1216 (93.9)	79 (6.1)	0.78	0.57–1.07	**0.64**	**0.42–0.97**
**Electronic media communication with parents**						
Often (reference group)	967 (91.7)	87 (8.3)	1.00		1.00	
Rare	1306 (94.2)	80 (5.8)	**0.68**	**0.50–0.93**	**0.47**	**0.32–0.69**

^a^ Method = Enter. Significant relationships are provided in bold. OR—Odds Ratio; 95% CI—95% Confidence Interval.

## Abbreviations

The following abbreviations are used in this manuscript:

BLR	Binary logistic regression
CI	Confidence interval
FAS	Family affluence scale
HBSC	Health Behaviour in School-aged Children study
OR	Odds ratio
